# The contribution of matrix metalloproteinases and their inhibitors to the development, progression, and rupture of abdominal aortic aneurysms

**DOI:** 10.3389/fcvm.2023.1248561

**Published:** 2023-09-19

**Authors:** Georgia Atkinson, Rosaria Bianco, Karina Di Gregoli, Jason L. Johnson

**Affiliations:** Laboratory of Cardiovascular Pathology, Department of Translational Health Sciences, Bristol Medical School, University of Bristol, Bristol, United Kingdom

**Keywords:** aortic aneurysm (AA), matrix metalloproteinase (MMP), animal models, TIMP (tissue inhibitors of metallproteinase), VSMC = vascular smooth muscle cell, macrophage

## Abstract

Abdominal aortic aneurysms (AAAs) account for up to 8% of deaths in men aged 65 years and over and 2.2% of women. Patients with AAAs often have atherosclerosis, and intimal atherosclerosis is generally present in AAAs. Accordingly, AAAs are considered a form of atherosclerosis and are frequently referred to as atherosclerotic aneurysms. Pathological observations advocate inflammatory cell infiltration alongside adverse extracellular matrix degradation as key contributing factors to the formation of human atherosclerotic AAAs. Therefore, macrophage production of proteolytic enzymes is deemed responsible for the damaging loss of ECM proteins, especially elastin and fibrillar collagens, which characterise AAA progression and rupture. Matrix metalloproteinases (MMPs) and their regulation by tissue inhibitors metalloproteinases (TIMPs) can orchestrate not only ECM remodelling, but also moderate the proliferation, migration, and apoptosis of resident aortic cells, alongside the recruitment and subsequent behaviour of inflammatory cells. Accordingly, MMPs are thought to play a central regulatory role in the development, progression, and eventual rupture of abdominal aortic aneurysms (AAAs). Together, clinical and animal studies have shed light on the complex and often diverse effects MMPs and TIMPs impart during the development of AAAs. This dichotomy is underlined from evidence utilising broad-spectrum MMP inhibition in animal models and clinical trials which have failed to provide consistent protection from AAA progression, although more encouraging results have been observed through deployment of selective inhibitors. This review provides a summary of the supporting evidence connecting the contribution of individual MMPs to AAA development, progression, and eventual rupture. Topics discussed include structural, functional, and cell-specific diversity of MMP members; evidence from animal models of AAA and comparisons with findings in humans; the dual role of MMPs and the requirement to selectively target individual MMPs; and the advances in identifying aberrant MMP activity. As evidenced, our developing understanding of the multifaceted roles individual MMPs perform during the progression and rupture of AAAs, should motivate clinical trials assessing the therapeutic potential of selective MMP inhibitors, which could restrict AAA-related morbidity and mortality worldwide.

## Introduction

Abdominal aortic aneurysms (AAA) demonstrate a major, life-threatening cardiovascular condition as demonstrated through mortality rates associated with ruptured AAAs estimated at 90% (1). Progress has been made in elucidating the multifactorial process of AAA pathogenesis, in particular the components of chronic inflammation which has led to promising candidates for conservative treatments currently being tested in several prospective clinical trials. Yet to date, surgical repair remains the only curative approach, but is associated with considerable morbidity and mortality rates.

Matrix metalloproteinases (MMPs) are a family of enzymes which degrade major components of the vessel wall. Additionally, resident and recruited cells can utilise MMPs directly and indirectly to facilitate their behaviour, including proliferation, migration, and survival. As a result, MMPs intimately contribute to the development and progression of multiple cardiovascular diseases, including AAA. An imbalance in the ratio between MMPs and the endogenous tissue inhibitors of MMPs (TIMPs) is believed to be a critical part of the dysregulated extracellular matrix degradation seen in AAAs, with numerous studies observing increased MMP activity in AAA patients (2). This review provides an overview of the role of MMPs and TIMPs in AAA development, progression, and rupture.

## Abdominal aortic aneurysm

### Anatomical sites of aneurysm

Aortic aneurysms are principally found within the abdominal and thoracic aorta and are respectively referred to as AAA and TAA. AAAs are the most common as data revealed a prevalence of 5%–6% for men and 1%–2% for women, in people older than 65 years, in comparison to a prevalence of five-fold lower for TAA (3). TAAs can be observed in different aortic segments including the aortic root, ascending aorta, aortic arch, or descending aorta. Sixty percent of TAAs develop in the aortic root and ascending aorta, forty percent are attributed to the descending aorta, whereas only ten percent involve the arch and the thoracic-abdominal area (4). AAAs are characterised as suprarenal when located within the suprarenal region of the abdominal aorta where the visceral arteries are involved. They are defined as juxtarenal (also termed pararenal) if the aneurysm extends up to but does not involve the renal arteries, or as infrarenal if they begin 10 mm below the renal arteries (5, 6). The pathogenesis of AAAs and TAAs is distinct.

### Aetiology and clinical complications

AAAs have a complex multifactorial pathogenesis in which genetic and environmental risk factors play a prominent role (5). Clinically, there is no universal definition of AAA, however, it is widely accepted to be a maximum infrarenal abdominal aortic diameter of >30 mm, measured using ultrasonography or computed tomography angiogram (CTA). Although the exact cause of AAAs is unknown, several factors are considered to contribute to the focal weakening of the aortic wall, increasing an individual's risk of developing an aneurysm.

Males are around four-times more likely to be diagnosed with an aneurysm during their lifetime compared to females, with this risk further increased by 40% every five years after the age of 65 (7). Moreover, AAA incidence is higher among the Caucasian population compared to those of African-American, Asian and Hispanic descent (8). Smoking has been highlighted as a major risk factor for aneurysm initiation and progression (9). The deleterious effects of smoking have been attributed to alterations in the inflammatory response of the aortic wall, alongside increased matrix degradation due to an imbalance between proteases (such as matrix metalloproteinases) and protease inhibitors. Moreover, there is an association between smoking duration and number of cigarettes smoked with AAA risk. Other risk factors include a family history of aneurysms. In particular, it has been shown that individuals with a first-degree relative with an AAA have a 30% higher risk of developing an AAA themselves as well as a higher risk of aneurysm rupture and increased likelihood of developing an aneurysm at a younger age (10). Another study revealed that the growth rate of aneurysms is doubled in patients with familial history when compared to patients with no evidence of previous familial disease (11).

AAAs often grow slowly and are typically asymptomatic until they rupture, making them difficult to detect. This is the main complication of AAAs and underlies their high mortality rates of approximately 60%–80%. However, when symptoms do arise, these usually display as constant abdominal or back tenderness or pain, which can last for hours up to days. Some patients report a palpable abdominal mass. At times, AAAs may cause symptoms because of local compression, leading to early satiety, nausea, vomiting, urinary symptoms, or venous thrombosis due to venous compression. Aneurysms that produce symptoms are at an increased risk for rupture, leading to further increased mortality rates. Due to the asymptomatic nature of most AAAs, diagnosis commonly results from incidental abdominal screening or during a routine examination involving abdominal palpation (12). Nationwide screening programmes have been implemented in several countries with the aim to alleviate AAA-specific mortality. Ultrasonography and computed tomography angiography are first-line imaging tools to identify and manage AAAs (13). An ultrasound scan is an accurate method to measure aneurysm size, with the benefit of being inexpensive and non-invasive. Unfortunately, many AAAs remain undetected and a ruptured aortic aneurysm can lead to life-threatening internal bleeding, with larger aneurysms exhibiting a greater risk (14, 15).

### Current therapies

Therapies for AAAs depend on a variety of factors such as location and size, as well as patient-specific factors like age, risk factors, or other existing conditions that may increase the risk during surgical intervention. Small aneurysms (less than 5.5 cm in diameter) are deemed at a low risk of rupture and are typically treated with pharmacological interventions to control blood pressure, aiming to reduce shear stress within the aorta (15). For example, beta-blockers may decrease the rate at which aneurysms grow by reducing blood pressure and contractility of the left ventricle (15). Statins have also been considered as a good therapy due to their pleiotropic anti-inflammatory properties, as well as their ability to reduce the expression of MMPs (16). Similarly, angiotensin-converting enzyme (ACE)-inhibitors and Ang II receptor blockers (ARBs) have been proposed, and are already widely deployed for the treatment of other cardiovascular diseases (15, 16). Additionally, antibiotic therapy has been evaluated as a method to reduce aneurysm expansion by reducing elastin degradation and inflammation (17). Finally, lifestyle changes are recommended to improve outcomes and prevent aneurysm growth and rupture. As mentioned above, smoking is a risk factor of aneurysm growth and rupture, therefore the American College of Cardiology and American Heart Association have advised those with a positive familial history of AAA to stop smoking (18). However, there is currently no pharmacological therapy established to slow or prevent AAA growth and rupture directly.

Accordingly, two main methods of elective intervention are used as surgical options and are the only available curative treatment for AAA: open surgical repair and endovascular aneurysm repair (EVAR). Usually, surgical treatment is adopted only when the diameter of the aorta is greater than 5.5 cm, as risk of rupture outweighs the risk of surgical complications. Open surgical repair involves the removal of the damaged section of the aorta and replacement with a synthetic vascular graft. This will allow blood to flow through the graft, bypassing the aneurysmal area and therefore, reducing pressure on the damaged wall of the aorta. This procedure requires a 2–3-month recovery and is associated with a high risk of mortality (19). Alternatively, EVAR involves the minimally invasive insertion of a synthetic graft within the aneurysm via groin incisions to access the femoral artery. This is considered a less invasive procedure due to less interference with the circulation, resulting in a reduced recovery period. Considering outcomes, a study comparing both short-term and long-term survival of patients undergoing either procedure revealed that mortality rates are similar and inﬂuenced by age, co-morbidities, and pharmacological interventions (20).

### Pathogenesis and composition

**Figure 1:**
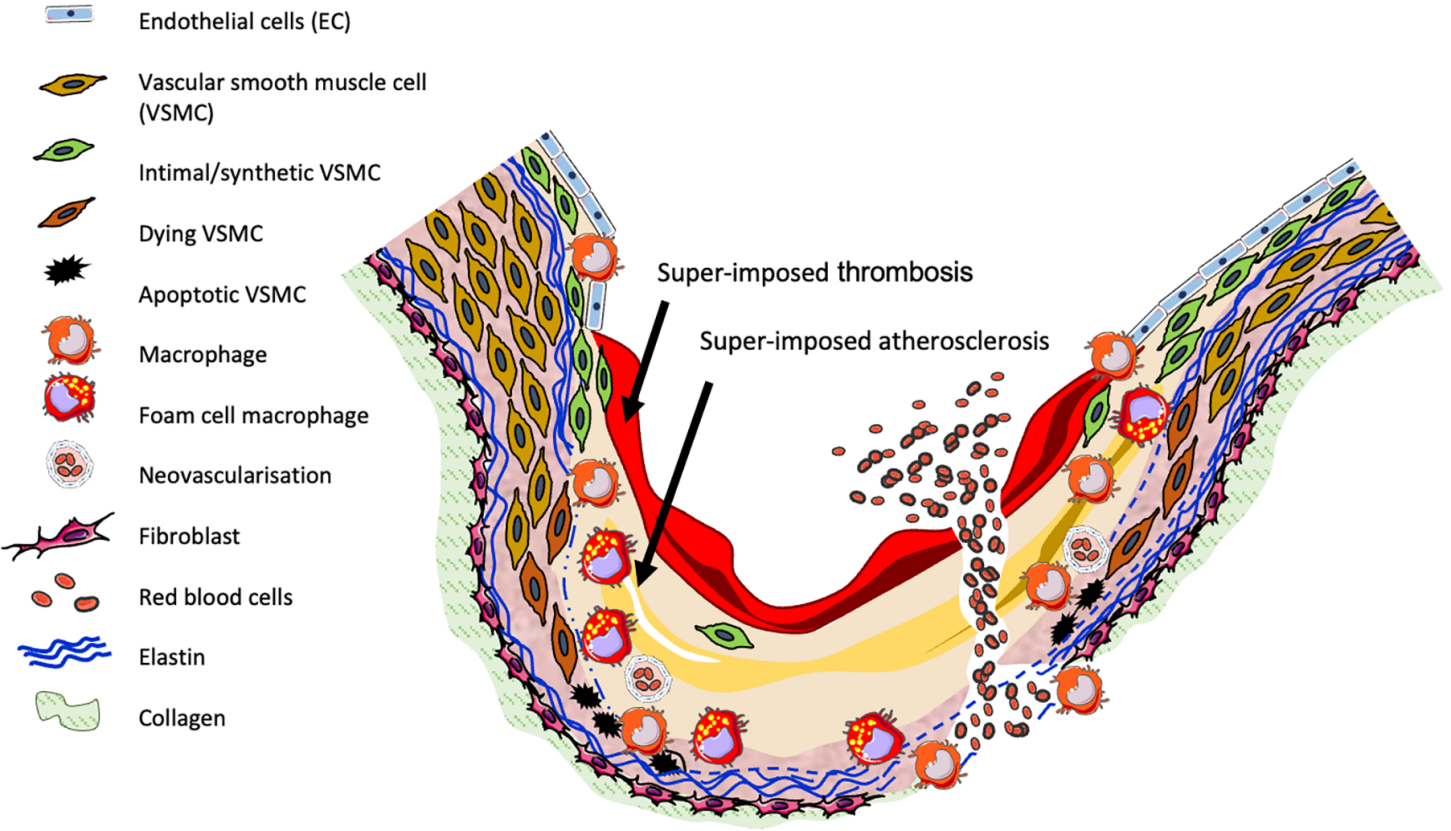
Diagram of human AAA pathogenesis.

As shown in Figure 1, human aneurysms are characterised by structural deterioration of the aortic wall, consequent progressive aortic dilatation and ultimately rupture. Multiple factors are involved in the pathogenesis of AAA which cause focal destructive remodelling of connective tissue through all layers of the aortic wall. A major pathobiological aspect of AAA includes loss of key extracellular matrix (ECM) proteins through the proteolytic degradation of collagen and elastin, accompanied by the infiltration and accumulation of inflammatory cells throughout all layers of the aortic wall, alongside resultant secretion of inflammatory growth factors and cytokines. Additionally, AAAs are commonly characterised by a reduction in VSMC content due to apoptosis, and the presence of neovascularisation (1, 21). MMPs and other proteases are produced by macrophages and VSMCs and are proposed to contribute to many of the above processes.

### Collagen and elastin

Collagen and elastin are the most abundant extracellular matrix proteins found within large and mid-sized arteries, imparting the elastic properties, structural strength and mechanical resistance to the vessel wall (22). Collagen fibrils are stable structures formed from the aggregation of several subunits, called tropocollagen, which play an essential role in the artery wall providing the necessary strength and stability. Twenty-nine types of collagen have been identified in the human body, although 80%–90% of collagen present in the body is of type I, II, and III (1, 23). Within the aortic wall, type I collagen accounts for 70% of the total collagen content, and is located predominantly in the intima and adventitia, whereas type III collagen is expressed within the media and along the elastic lamina (24). Several studies have identified collagen loss within aneurysmal aortae, contributing to the instability of the aortic wall. ECM degradation is prevalent during aneurysm progression, because of increased expression and activity of proteases, especially MMPs, leading to increased collagen degradation (25, 26). Accordingly, the combination of decreased vascular smooth muscle cell (VSMC) collagen synthesis (25) alongside enhanced degradation highlights the major contribution attributed to collagen turnover during aneurysm progression. Accordingly, products released by collagen catabolism have been indicated as potential aneurysm biomarkers, especially the amino terminal pro-peptide of type III procollagen (PIIIN). In AAA patients, serum levels of PIIIN were significantly elevated compared to control subjects in two separate studies, however the serum level of the carboxyterminal pro-peptide of type I procollagen (PICP) was similar between groups within multiple studies (27).

Elastin is organised as fibres within the medial portion of the aortic wall. The interaction of these fibres with VSMCs and collagen provide a solid structure which is necessary for the biomechanical and elastic properties of the aorta (28). Alterations in elastin fibre quantity alongside variations in collagen structure underlie the mechanical and functional changes related to aortic diseases. Specifically, elastin degradation occurs during aneurysm progression, releasing elastin-derived peptides into the circulation. A screening study revealed that patients with a ruptured AAA greater than 6 cm display increased levels of elastin-derived peptide, compared to those with small aneurysms that have not ruptured (29). The primary catabolic enzyme responsible of elastin degradation is elastase, a family of eight human genes including chymotrypsin (CTRC), neutrophil elastase (ELANE), and MMP-12 (also known as macrophage metalloelastase) (30), whose activity is inhibited by endogenous protease inhibitors such as alpha-1 anti-trypsin (A1AT) (31) and TIMP-3 (32). It has previously been shown that serum levels of A1AT are higher in AAA patients than those with aortic-occlusive disease, highlighting the role of elastin degradation during aneurysm progression and the response to alleviate increased elastase activity (1). As discussed in greater detail later, researchers have exploited the proposed central role of elastase in AAA development and progression through deploying intra-aortic elastase perfusion to induce AAA formation in mice, rats, rabbits, dogs, and pigs (33). The utility of this experimental approach demonstrated that elastase activity contributed to aneurysm formation and corroborated quantitative correlation between elastase concentration and aneurysm size (34). In summary, alterations in the structure of elastin and collagen contributes towards aneurysm formation and progression. Moreover, aortic dilation is linked with elastin loss, whereas aortic rupture is associated with loss of collagen (22, 35).

### Vascular smooth muscle cells

VSMCs are the most abundant cell type within the blood vessel wall, and play an important role in the structure of aorta wall by maintaining production of elastin, collagen, matrix proteins, several proteases, and inhibitors (36). VSMCs regulate the contractile tone of the vessel alongside the secretion of ECM proteins which highlights the two different VSMC phenotypes, synthetic and contractile (37). In healthy blood vessels, the predominant phenotype present is the contractile form, due to the limited secretion of ECM proteins and profuse myofilament production to allow regulation of blood flow and vessel diameter. During pathological conditions, the switching of VSMC phenotype from contractile to synthetic is induced, which is characterised through a decrease in contractile protein expression alongside an increased production of proteases such as collagenolytic and elastinolytic MMPs, which can degrade elastin and collagen and promote vessel dilation, weakening, and eventual rupture (21, 38).

Together with VSMC phenotypic modulation, a reduced number of VSMCs is observed in AAAs (39), attributed to their enhanced susceptibility to apoptosis, as VSMCs undergoing apoptosis are observed within the medial portion of human AAAs (40). Multiple factors have been proposed to induce VSMC apoptosis including oxidised lipoproteins, cytokines, and increased reactive oxygen species (41), alongside extracellular matrix degradation (42) and mechanical stress (43). Angiotensin II can also trigger VSMC apoptosis through ligation of angiotensin receptor types 1 and 2, with angiotensin II infusion shown to induce AAA formation in mice (33) which is associated with medial VSMC apoptosis (44). It was also found that VSMCs within aneurysms showed increased production and accumulation of p53, a pro-apoptotic protein and a member of the Bcl-2 protein family (39), a mechanism corroborated in a mouse elastase-induced aneurysm model (45).

### Calcification

Abdominal aortic calcification (AAC) has been observed as a sign of a degenerative inflammatory process within the arterial wall, occurring at two distinct sites: the intima and the media. Mainly, intimal calcification is observed in advanced atherosclerosis, with many studies revealing an association between calcification and risk of cardiovascular events, supporting its potential as a biomarker (46, 47). Calcification is characterised by lipid deposition, macrophage infiltration and VSMC proliferation, whereas medial calcification can exist independently of atherosclerosis and is usually associated with elastic fibres (48). Arterial calcification causes a reduction in the elasticity of the vessel wall which increases the risk of rupture during aneurysm development and progression (49, 50). A proteomics study of calcified AAAs (CAAs) revealed type I collagen α-1 and type III collagen α-1 chains were increased in CAA, whereas type XIV collagen α-1 was decreased compared to healthy controls (51). Furthermore, levels of ECM proteins such as fibulin-5 were decreased in calcified TAA (CTA) patients (51). Fibulin-5 plays an important role in endothelial cell adhesion and elastin fibre integrity, and is therefore important in preserving the stability of the aorta wall, through its integrin-binding matricellular protein properties (52).

### Angiogenesis/neo-vascularisation

Angiogenesis, characterised by the formation of new blood vessels from pre-existing vessels, plays an essential role in many developmental and pathological processes, including embryogenesis, inflammation, tissue development and repair (53). The induction of angiogenesis begins with ECM degradation and activation of vascular endothelial cells, with their increased proliferation and migration essential for new blood vessel formation. In healthy individuals, the balance of pro- and anti-angiogenic factors is maintained, whereas in pathological conditions there is an imbalance which favours the accumulation of pro-angiogenic factors (54). MMPs have been recognised as a factor strongly involved in angiogenesis, and a histological study of human AAAs demonstrated prevalent angiogenesis (also termed neovascularisation) within the media of the aortic wall which associated with increased MMP expression and activity (55), especially ruptured AAAs (56). Accordingly, neovascularisation within AAAs co-localise with areas of elastin degradation and macrophage accumulation, which are mainly restricted to the outer medial aspect and remodelling adventitia (53, 57).

### Inflammation

Inflammation has been postulated to play a pivotal role in the pathogenesis of aneurysm. Inflammatory cells including neutrophils, T cells, B cells, macrophages, mast cells and NK cells can permeate through the aortic wall and are associated with increased production of pro-inflammatory cytokines and chemokines (58). Furthermore, VSMCs, endothelial cells and monocyte/macrophage MMP expression can be regulated by chemokines and lead to ECM degradation, contributing to the weakening of the aortic wall during aneurysm progression (59, 60). Cytokines are intercellular messenger proteins involved in the regulation of inflammation, haematopoiesis, cellular and humoral responses, and wound healing (60). Prevalent inflammatory cells found within aneurysms include cluster of differentiation 4+ (CD4+) T cells, B cells and macrophages. CD4 + type 1 T helper cells (Th1) and CD8+ T-cytotoxic type-1 (Tc1) cells produce interferon gamma (IFNγ), interleukin-2 (IL-2) and tumour necrosis factor (TNFα), whereas type 2 T helper cells (Th2) and type 2 cytotoxic cells (Tc2) secrete IL-4, IL-5, IL-10 and IL-13, which are also produced by B cells, macrophages, NK cells and ECs.

TNFα and IFNγ-driven inflammatory processes are prominent in the pathogenesis of aneurysms due its role in activation and recruitment of immune cells to inflammatory sites, alongside secretion of pro-inflammatory cytokines and MMPs, and inducing VSMC death (59, 61). Elevated levels of TNFα mRNA and protein were reported in aneurysmal tissue of rodent models, and elastase levels reduced after delivery of a TNFα binding protein, which suggests that TNFα may play a role in the pathogenic mechanisms of AAA (62). Similarly, increased levels of IFNγ have been observed in human AAA compared to controls and has been shown to suppress VSMC collagen production (63). IFNγ also stimulates macrophage and VSMC MMP production, further contributing to aneurysm formation (59). However, there is conflicting evidence over the role of this cytokine in AAA pathogenesis, as conversely, studies have found that blocking the IFNγ receptor led to accelerated aneurysm formation (64). This may be due to the divergent roles of IFNγ, which can exert both anti- and pro-inflammatory effects, dependent on the pathology and cytokines involved.

Transforming growth factor-β (TGFβ) can modulate the structure and composition of the ECM and therefore considered an important regulator of vascular remodelling. TGFβ is produced by multiple cell types and participates in a wide array of cellular responses including proliferation, angiogenesis, differentiation, apoptosis, inflammation, and wound healing. Reduced TGFβ levels have been detected in AAA patients, which correlated with reduced cystatin C, a cysteine protease inhibitor (65). TGFβ can also retard MMP-12 expression and therefore abrogate aneurysm progression in several mouse models (66). However, similarly to IFNγ, divergent roles for TGFβ have been proposed, with increased TGFβ activity shown to associate with AAA formation, and subsequent functional blocking of TGFβ reduced aneurysm formation (67, 68).

In addition, other cytokines such as macrophage migration inhibitory factor (MIF) have been implicated in aneurysm pathogenesis. MIF functions as a pleiotropic protein, participating in inflammatory and immune responses, in particular the stimulation of angiogenesis and regulation of proliferation (69). MIF also regulates the production and activation of T and B cells as well as modulating proteolysis through the regulation of MMP expression, and activation of urokinase plasminogen activator (uPA) and tissue plasminogen activator (tPA) (61). Granulocyte/macrophage-colony stimulating factor (GM-CSF) regulates monocyte production and maturation, while also polarising macrophages towards a pro-inflammatory phenotype with heightened expression of MMPs (70). GM-CSF administration to hypercholesterolaemic mice was shown to accelerate AAA formation (71), while GM-CSF inhibition dampened AAA development in mice which was associated with decreased inflammation and related MMP expression/activity, and elevated GM-CSF expressing inflammatory cells were detected within human AAA samples compared to non-diseased aortic samples (72). These findings further support a direct role for inflammation in the pathogenesis of AAA and highlight GM-CSF as a potential therapeutic target.

Relatedly, C-reactive protein (CRP) is considered a biomarker of cardiovascular events and an indicator of systemic inflammation. A study by Vainas and colleagues found high levels of serum CRP in patients with an increased aortic diameter (73). Patients with symptomatic or ruptured AAA showed higher circulating levels of CRP and this correlated with an increased aneurysm size. This relationship between CRP and aneurysm size was further confirmed in a study that demonstrated that the CRP level was greater in patients with large AAAs, compared to small ones, and a marked difference between AAA patients and healthy controls (74), further qualifying a central role for inflammation in AAA development and progression.

### Contribution of atherosclerosis

Atherosclerosis is characterised by thickening of the arterial wall due to the accumulation of lipids, vascular and inflammatory cells, and the deposition of various ECM proteins within the tunica intima (75). Several studies have revealed a strong association with atherosclerosis and aneurysm formation, with atherosclerosis commonly being considered a risk factor for subsequent aneurysm formation (76). However, it is still unclear if these pathologies are linked due to common risk factors or exert direct causal effects (77). In the past, different concepts have been developed to explain the relationship between AAA and atherosclerosis, with the general consensus being that AAAs are caused by atherosclerosis and are therefore called atherosclerotic aneurysms typically occurring within the abdominal aorta (78). Indeed, the pathophysiology of AAA has been termed as a highly proteolytic from of atherothrombosis (79). It has been proposed that luminal narrowing of the aorta, typical of atherosclerosis formation, induces modification and remodelling of the vascular wall to compensate for the loss of lumen patency, involving ECM remodelling to try and correct the vessel diameter. However, this inadvertently induces medial thinning and therefore weakening of the vessel wall, typical of AAA development (80). It is also plausible that AAA and atherosclerosis are independent pathologies with different aetiologies but are strongly linked with common risk factors such as smoking, hypertension and hypercholesterolaemia, although as discussed above, human AAAs harbour overlying atherosclerotic lesions. A final concept suggests that AAA or aortic atherosclerosis can develop first and consequently encourage development of the other. A study conducted by Golledge et al. stated that several independent mechanisms are responsible for AAA and atherosclerotic plaque formation which are different from patient to patient, concluding that it is important to avoid broad standardised therapeutics that would not be successful (80).

### Dissection vs. aneurysm

Aortic dissection is a lethal condition in which the inner layer of the aorta tears causing blood to push through the tear, separating the middle layer of the wall from the outer layer, creating a new lumen (false lumen). In some cases, the dissection will cross all three layers of the aortic wall and cause immediate rupture and almost certain death (81, 82). Several conditions can alter and weaken the aorta wall, subsequently predisposing individuals to an aortic dissection. These include hypertension, atherosclerosis, disorders of the connective tissue (Marfan Syndrome, Ehlers-Danlos Syndrome), genetic disorders or trauma, which can cause damage to the aorta wall, so as to cause an aortic dissection (83). Conversely, an aneurysm involves focal dilatation of a blood vessel, which may become weakened and subsequently rupture and cause death (84). Common risk factors for both aneurysm and dissection include ageing, atherosclerosis, and hypertension (85). Moreover, if an individual develops an AAA, this becomes a strong risk factor for future aortic dissection. In addition, once an aortic dissection occurs, the aorta becomes weak and may enlarge over time, which may ultimately lead to the development of an aneurysm. Therefore, although aneurysms and dissections have separate pathologies and aetiologies, they can be associated.

### Animal models of AAA

AAA is a common degenerative disorder associated with aortic rupture and consequently sudden death, with current therapies being limited. Accordingly, several animal models have been developed with the purpose of further elucidating the pathogenesis of AAA and illuminate the biochemical and cellular mechanisms underlying this disease (86). A wide range of animal models have been developed across a variety of species including pig, sheep, dogs, rabbits, rodents, and primates. Like humans, certain dog breeds have comparable artery size as well as ability to endure prolonged anaesthesia. Pigs also display a high level of similarity in the morphology of the arterial system compared to humans, and the coagulation process is comparable between sheep and humans. Finally, primates have a high level of similarity with humans, especially the fibrinolytic system. However, costs and ethical concerns related to the use of these large animal species restrict their utilisation in aneurysm studies.

**Figure 2:**
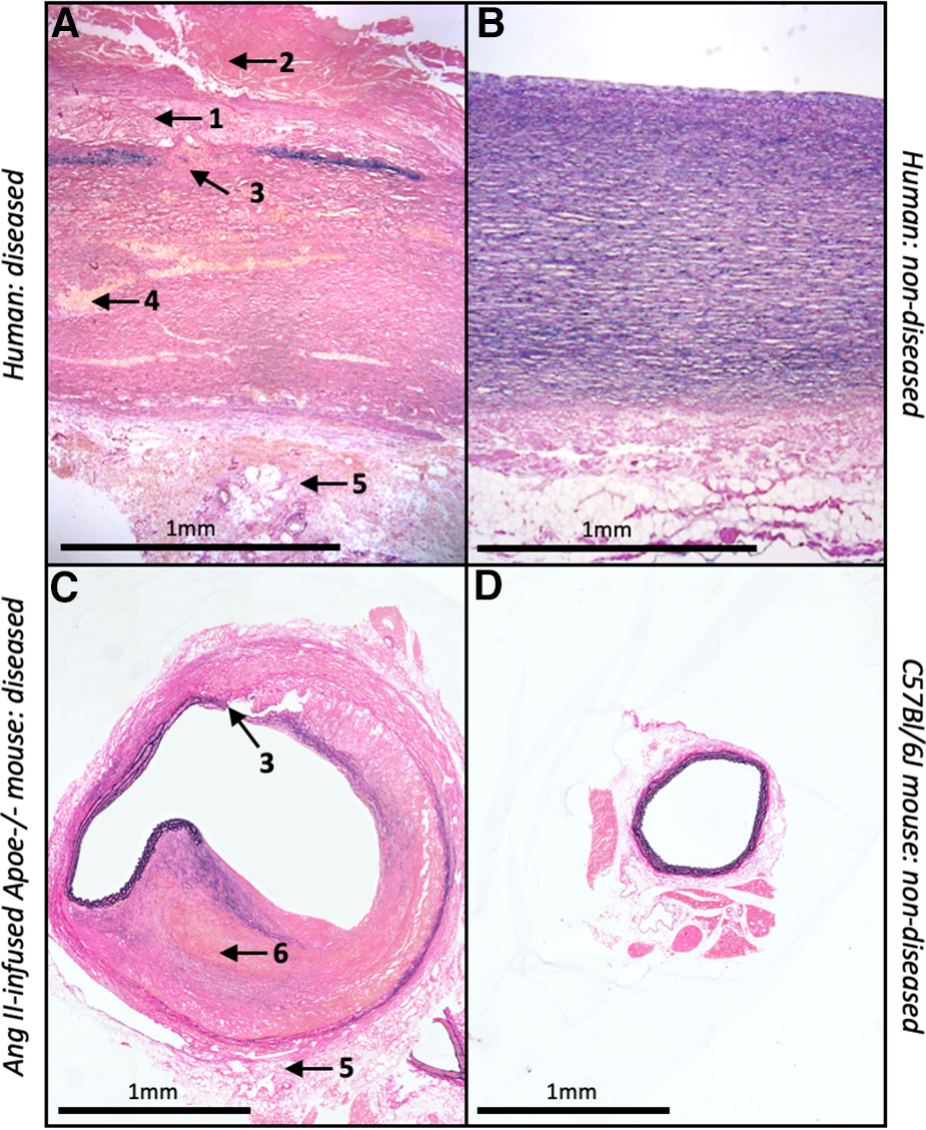
Similarities and differences between human and mouse AAAs. Histological images of EVG-stained abdominal aortae from (**A**) diseased and (**B**) non-diseased humans alongside (**C**) Ang II-infused Apoe^-/-^ mouse and (**D**) C57Bl/6J wild-type mouse. Numbers and associated arrows indicate the following histological characteristics; 1 = atherosclerosis; 2 = intra-luminal thrombus; 3 = medial degeneration; 4 = VSMC loss; 5 = adventitial remodelling; 6 = intra-mural thrombus.

Accordingly, the mouse is a desirable species to model AAA due to their small size, relatively low costs and their capacity to overexpress or delete specific genes (87). There are several classes of experimental AAA which can be divided in three categories: genetically predisposed animal models, chemical and physical models (33). The most commonly used approach is chemical induction and includes methods such as the localised perfusion of elastase, application of calcium chloride to the adventitia of the aorta, or systemic infusion of angiotensin-II (88). Most published studies utilised the angiotensin II (Ang II)-infusion mouse model which involves the subcutaneous implantation of osmotic mini-pumps in male and/or female apolipoprotein E-deficient (Apoe-/-) mice to deliver Ang II (500–1000 ng/kg/min). This induces suprarenal aneurysm formation, approximately 3–15 days after implantation with a reported mortality rate of 15%–30% attributed to aortic rupture (89, 90). Although rarely reported in mouse AAA studies, early atherosclerotic plaque formation can be induced in the Ang II-infusion mouse model when combined with high-fat feeding. The aneurysms which from within the suprarenal abdominal aortae of Ang II-infused Apoe-/-mice are characterised by medial degeneration, inflammation, and intra-mural thrombosis (89). Furthermore, although Ang II is a hypertension-inducing molecule, blood pressure assessment in Apoe-/-mice revealed that aneurysm formation in this model was independent of blood pressure changes (88). However, it should be noted that ultra-high-resolution imaging studies have suggested that Ang II-infusion induces rupture of the aortic medial layer which results in formation of an intra-mural haematoma/thrombus and subsequent partial dissection of the adventitia within the supra-renal region of the abdominal aorta (91). Accordingly, it has been proposed that the Ang II-infusion Apoe-/- mouse may model aortic dissection rather than fusiform AAA-related rupture, with mice displaying intra-mural rather than intra-luminal thrombus, which is prevalent in most human AAAs (92); these disparities are highlighted in Figure 2.

Adventitial application of calcium chloride solution is another method adopted to induce aortic aneurysm formation. This method was first applied to rabbit aortas, and subsequently mice, involving the application of 0.25–1 molar calcium chloride to the adventitia of the infra-renal abdominal aorta, using either a soaked swab or directly in solution (93). Results illustrated an increase in the aorta diameter by 64% after two weeks of application and by 110% after three weeks, alongside a marked inflammatory response (94). Further features of this model that correlate with human AAAs include medial calcification, elastin loss, VSMC apoptosis, and increased proteolytic activity (33). The elastase infusion model is further model deployed in AAA studies. This method requires the introduction of a catheter into the infra-renal aorta and isolation of a segment of the abdominal aorta by distal suture before perfusion with elastase, commonly porcine pancreatic elastase (34). This leads to medial destruction, dilatation of the aorta and consequently, aneurysm formation within 2–4 weeks after the procedure in rats and mice (86).

## Matrix metalloproteinases (MMPs) and tissue inhibitors of MMPs (TIMPs)

### MMPs—structure, classification, activity and role in inflammation and vascular remodelling

**Figure 3:**
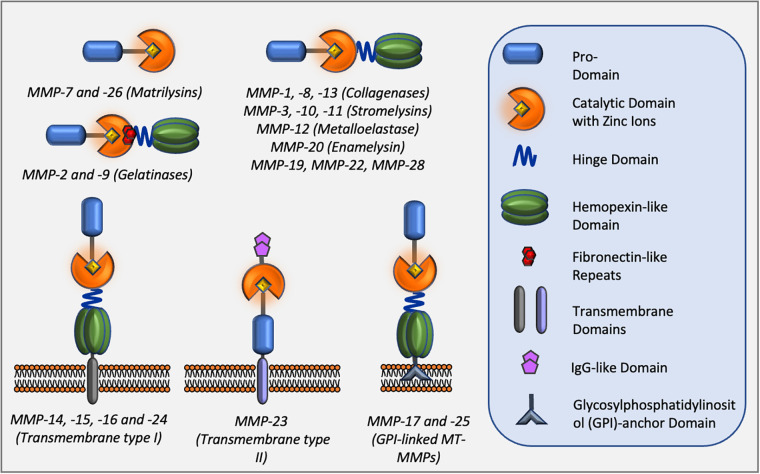
Classes of MMPs and their domain structure.

Matrix metalloproteinases (MMPs), also known as matrixins, are a family of proteolytic enzymes capable of degrading many components of the extracellular matrix (ECM) (2). MMPs are involved in a multitude of physiological processes such as morphogenesis, tissue repair, and remodelling. However, they also play a crucial role in pathological events including many cardiovascular diseases such as atherosclerosis, post MI remodelling, and aneurysm formation, progression, and rupture (26). In addition to degradation of ECM components, MMPs have the ability to target and process non-ECM molecules directly affecting cell behaviour (95). Consequently, MMP activity has been linked to migration/invasion, proliferation, and apoptosis of several component cells of the blood vessels, VSMCs, endothelial cells, and monocyte/macrophages (96). The MMP family consists of 23 members which share structural similarities and are included in the Metzincin family together with ADAMs (a disintegrin and metalloproteinase family) and ADAMTs (ADAM with thrombospondin motifs) proteases (97). The structure and classification of MMPs are summarised in Figure 3. MMPs are produced as latent enzymes (pro-forms) and therefore they require activation through pro-domain cleavage. Stepwise activation of secreted MMPs require the destabilisation, followed by full cleavage of the pro-domain by either plasma, bacterial protease and/or, in some cases, by other active MMPs (97). Membrane type-1 and type-2 MMPs (MT-MMPs) are fully activated intracellularly by furin or other pro-protein convertases before being expressed on the cell membrane as active enzymes. Similarly, MMP-11 and -28 are intracellularly activated by furin and secreted as active enzymes into the extracellular space.

Due to the potential damaging actions of uncontrolled MMP expression, their activity is tightly regulated by multiple endogenous protease inhibitors; these include α2-macroglobulin, the reversion-inducing cysteine-rich protein with Kazal motifs (RECK), tissue factor pathway inhibitor-2 (TFPI-2), and the pro-collagen C-terminal proteinase enhancer (PCPE) (97). However, tissue inhibitor of MMPs (TIMPs) are the most potent endogenous inhibitors of MMPs, playing a major role in regulating MMP activity during physiological and pathobiological processes (98). The balance between MMPs and TIMPs is crucial for homeostasis and dysregulation can result in pathological dysfunctions associated with an aberrant turnover of the extracellular matrix and/or alterations in cell behaviour. Four different TIMPs have been identified in vertebrates (TIMP-1, -2, -3, and -4); they are secreted proteins able to form tight complexes with MMP catalytic domains due to inhibitory residues existing within their N-terminal domain. Each distinct TIMP exhibits diverse inhibitory efficacy against different members of the MMP family (i.e., TIMP-1 has a poor inhibitory effect on MMP-9, -14, -15, -16, and -24) (96). Moreover, TIMPs also retain the ability to inhibit members of both the ADAM and ADAMTS family of proteinases (96) which, however, will not be discussed in the present review. Finally, TIMP-3 is distinct to the other TIMPs as it displays high-affinity for extracellular matrix proteins through interactions with its C-terminal domain, facilitating TIMP-3 accumulation within the matrix and subsequently extending its half-life (98).

MMPs share structural homology and are classified into six classes based upon their structure. All members contain a pro-domain (blue) which requires removal during activation. A linker hinge domain separates the catalytic domain (orange) from the hemopexin-like domain (green) for members of the collagenase and stromelysin families. Three fibronectin-like repeats in the catalytic domain (red) are present within members of the gelatinase sub-family. Membrane-type MMPs also contain a transmembrane domain and a cytoplasmic tail which is linked to the cell membrane. Two members of membrane-type MMPs are glycosylphosphatidylinositol (GPI)-anchored MMPs.

MMPs have been proposed to play a significant role during inflammatory responses, as they can promote accumulation of inflammatory cells within areas of injury/damage through enabling their invasion, proliferation, and susceptibility to apoptosis. For instance, MMP-12 contributes to the invasion of monocyte/macrophages and their subsequent accrual at sites of inflammation including atherosclerotic plaques, attributed to the potent elastinolytic activity of MMP-12 (99, 100, 101). Similarly, MMP-14 has been shown to promote monocyte invasion through a synthetic matrix, and monocyte/macrophage accumulation at sites of sterile inflammation (mouse model of granuloma formation), as well as within atherosclerotic lesions (102, 103). Additionally, it has been indicated that TIMP-2 and TIMP-3, but not TIMP-1, can inhibit macrophage apoptosis by inhibiting MMP-14 and MMP-12 dependent cleavage of N-Cadherin, respectively (101, 103). Moreover, MMPs can modulate the bioavailability of inflammatory mediators either by modifying chemokines, cytokines, and growth factors, or by shedding membrane receptors (104). In particular, the MT-MMPs (such as MMP-14) are considered effective sheddases due to their pericellular location, and are afforded the capacity to control cell behaviour through modifying membrane protein localisation, including the cleavage of growth-factor and cytokine receptors, integrins, and proteins that regulate cell–cell contacts (105). These effects can be mediated by direct targeting of inflammatory molecules as well as indirect control of their activity through the modulation of other substrates, such as ECM proteins or co-receptors which are able to bind and maintain pro-inflammatory mediators in specific locations during inflammatory responses (106).

In addition to a prominent role in inflammation, MMP activity is also required for vascular remodelling, where degradation of ECM components is fundamental alongside substantial evidence indicating MMPs as modulators of VSMC migration, proliferation, and apoptosis. Heightened MMP-2 and MMP-9 expression/activity promotes VSMC migration and subsequent neointima formation in an *in vivo* model of carotid ligation-induced vascular injury (107, 108, 109), with a similar role proposed for MMP-3, ascribed to its regulation of MMP-9 activation (110). Regarding VSMC proliferation, conflicting results indicate a more complicated role of MMPs. Several MMPs are able to disrupt VSMC cell-cell contact through shedding of N-cadherin and subsequent activation of intracellular β-catenin signalling, successively promoting a pro-proliferative VSMC phenotype, particularly MMP-9 and MMP-12 (111). However, although Mmp9 deficiency resulted in retarded VSMC proliferation in response to arterial injury, but not in carotid ligation experiments (108, 109). Similarly, several studies utilising pharmacological broad-spectrum MMP inhibitors revealed ambiguous findings on VSMC proliferation (26). Whereas adenoviral-based gene transfer studies have shown that over-expression of individual TIMPs can abrogate adverse vascular remodelling and neointima formation, but with limited effects on VSMC proliferation (112, 113, 114, 115). MMP-9 has also been demonstrated to facilitate reorganisation of the ECM alongside its degradation, and therefore participated in adverse arterial remodelling in response to haemodynamic changes (116), with clear connotations for vessel expansion during AAA development. MMP activity can also mediate mobilisation of growth factors, such as fibroblast growth factor (FGF)-1 and FGF-2, potentially promoting VSMC proliferation (106). Together with migration and proliferation, VSMC apoptosis also contributes to vascular remodelling, and alongside MMP-12 and MMP-14, MMP-7 proteolytic activity has been linked to cleavage of N-cadherin in VSMCs promoting their apoptosis (117). Finally, dysregulated MMP activity can also modulate apoptosis through the processing death ligands (i.e., TNFα and Fas ligand) and/or their receptors, prompting apoptosis in an autocrine as well as paracrine manner (42).

### MMPs and TIMPs expression in human AAA—potential biomarkers of AAAs

Histological studies assessing the expression and activity of MMP family members and TIMPs in AAA tissues has demonstrated their spatial and temporal profile, including cell-type expression (Table 1). Most of the MMPs evaluated within AAAs are highly expressed by inflammatory cell infiltrates alongside VSMCs and endothelial cells. Inflammatory cells—which comprise of neutrophils, monocytes/macrophages, mast cells, B- and T-cells—may serve as a prominent source of MMPs in AAA, as their numbers increase during aneurysm progression through amplified cell recruitment and proliferation (135). The collagenase family members, MMP-1, -8, and -13 have been reported to be increased in macrophages and VSMCs within AAA tissues in comparison to healthy aortic tissue (136, 137), with MMP-2, -3, -9, -12, and -14 also displaying elevated expression (26). Additionally, genetic studies examining relationships between gene polymorphisms and AAA provide support for MMP-3 as an important contributory factor (138, 139). Spatially, MMP-2 expression and activity are co-localised primarily with VSMCs in human AAAs (140). Conversely, MMP-9 is predominantly expressed by macrophages, and its activity as assessed by zymography, is increased in ruptured aneurysms when matched to similar sized intact AAAs (140, 141). MMP-12 (also known as macrophage metalloelastase), is expressed by infiltrating-macrophages within the atherosclerotic portions and medial aspect of AAAs, which co-localise with areas of elastin fragmentation and loss (142). Furthermore, proteomics analysis of human AAAs confirmed increased accumulation of MMP-12 along with degradation of collagen XII, fibronectin, periostin, tenascin, and thrombospondin 2, and suggested MMP-12 as the dominant protease within human AAAs (143). Accordingly, MMP-12 is considered to have a direct role in the pathogenesis of the AAAs through its ability to preferentially degrade elastin, and facilitate macrophage invasion, two key characteristics of ruptured human AAAs. MMP-14, one of the most predominant membrane-bound MMPs in the vasculature, has also been detected in VSMCs within human AAAs (144). Lastly, transcriptomic analysis of monocyte-derived macrophages isolated from AAA and peripheral arterial occlusion patients revealed heightened MMP-27 levels in AAA individuals (145).

**Table 1:** MMP expression in human AAA tissues and plasma, alongside results of *in vivo* animal studies evaluating the effects of genetic deficiency for select matrix metalloproteinases (MMP) or tissue inhibitors of metalloproteinases (TIMPs) on AAA formation.

MMP#	Expression in human		Animal studies			Ref.
	AAAs	Plasma	Modulation	Model	AAA Development	
MMP-1	↑					
MMP-2	↑		Mmp2 ^−/−^	CaCl_2_	↓	(118)
			Mmp2 ^−/−^	CaCl_2 _+ BMT	No effect	(118)
MMP-3	↑		Mmp3 ^−/−^	Apoe ^−/−^ + HFD	↓	(119)
MMP-7	↑		Mmp7 ^−/−^	Ang II	No effect	(120)
MMP-8	↑					
MMP-9	↑	↑	Mmp9 ^−/−^	CaCl_2_	↓	(118)
			Mmp9 ^−/−^	CaCl_2 _+ BMT	↓	(118)
			Mmp9 ^−/−^	Elastase	↓	(121)
			Mmp9 ^−/−^	Elastase_ _+ BMT	↓	(121)
MMP-12	↑		Mmp12 ^−/−^	CaCl_2_	↓	(122)
			Mmp12 ^−/−^	Ang II + TGF*β* inhibition	↓	(123)
			Mmp12 ^−/−^	Elastase	No effect	(121)
			Mmp12 ^−/−^	Ang II + Apoe ^−/−^	↑	(124)
			Mmp12 ^−/−^	Ang II + PCSK9-AAV	↑	(124)
			Mmp12 ^−/−^^(mac−specific)^	Ang II + Apoe ^−/−^	↑	(124)
			MMP12 ^(mac over−expression)^	Carrageenan	↑	(125)
MMP-13	↑		Mmp13 ^−/−^	Elastase	↓	(126)
MMP-14	↑		Mmp14 ^−/−^^(mac−specific)^	CaCl_2_	↓	(127)
			Mmp14 SNP ^(Y573D)^	Ang II + PCSK9-AAV	↑	(128)
MMP-17			Mmp17 ^−/−^	Ang II	↓	(129)
TIMP-1	↓	↓	Timp1 ^−/−^	Apoe ^−/−^ + HFD	↑	(130)
			Timp1 ^−/−^	Elastase + HFD	↑	(131)
			Timp1 ^over−expression^	Xenograft	↓	(132)
TIMP-2	↓		Timp2 ^−/−^	CaCl_2_	↓	(133)
TIMP-3	↓	↑	Timp3 ^−/−^	Ang II	↑	(134)
			Timp3 ^−/−^	Ang II + Apoe ^−/−^ + HFD	↑	(32)
TIMP-4		↓				

↓, decreased; ↑, increased.

BMT, bone marrow transplantation; Apoe, apolipoprotein E; HFD, high-fat diet; Ang II, angiotensin II; CaCl2, calcium chloride; SNP, single nucleotide polymorphism; AAV, adeno-associated virus.

At the mRNA level, TIMP-1 (146) and TIMP-3 (32) were unchanged between AAA and non-aneurysmal tissues, although TIMP-2 levels were modestly increased within AAAs (146). When comparing human AAA samples to healthy aortae, although protein expression of TIMP-2 was decreased while TIMP-1 and TIMP-3 were increased, heightened activity of MMP-1, MMP-9, MMP-12, and MMP-14 were observed (147), and therefore credited with the excess degradation of collagen and elastin, and driving aneurysmal dilatation. Further supporting a protective role for TIMP-2, a promoter polymorphism (-418 G/C; rs8179090) that diminishes TIMP2 gene expression rate, has been identified as a risk factor of AAA (148). Although TIMP-3 expression is elevated in the plasma from AAA patients, it is considered a potential positive feed-back mechanism to negate increased MMP activity (145, 149, 150). Interestingly, macrophage TIMP-3 expression was decreased in human AAAs, independent of changes in mRNA levels, and associated with increased MMP activity (32). Although reduced plasma and tissue levels of TIMP-4 were reported in patients with ascending aortic aneurysmal patients compared to controls (151), expression in AAAs has not been examined.

In line with elevated tissue levels of select MMPs, several studies have ascertained MMP plasma levels in AAA patients alongside healthy controls or patients with aortic obstructive diseases, to determine if they may serve as biomarkers of AAA presence and progression (Table 1). Specifically, elevated MMP-9 plasma levels were detected in AAA patients compared to controls and, additionally, a reduction of plasma MMP-9 was observed after surgical repair of AAA (152, 153, 154). Moreover, MMP-9 plasma levels have also been associated with evidence of aortic medial remodelling and increased luminal diameter (155). Furthermore, MMP-1 alongside MMP-9 were identified as elevated in the plasma of patients with ruptured AAAs when compared with individuals harbouring intact AAAs, with MMP-9 levels also associating with 30-day mortality (156). However, no correlation between MMP-9 plasma levels and the yearly expansion of small diameter AAAs (35–49 mm) was observed, questioning the utility of MMP-9 as a plasma biomarker for AAA progression (157).

Single-cell transcriptomics have been deployed to examine the cell phenotype expression of genes during the development and progression of human and mouse aortic aneurysms including AAAs (158), with certain studies identifying changes in the expression of select MMPs and TIMPs. Focussing upon VSMC phenotypes, single-cell sequencing data analysis combined from two independent studies demonstrated that a “fibroblast-like” VSMC phenotype (characterised through expression of ACTA2, MYH11, COL1A1, COL1A2, and PDGFRA) was increased in human aortic aneurysm samples compared to normal aorta, and displayed augmented MMP2 expression compared to contractile VSMCs (159). In mice, two standalone studies both deploying the Ang II-infusion mouse model of AAA revealed a pro-inflammatory macrophage subpopulation (Netrin1 + ve) and VSMC Mmp3 levels are increased and contribute to AAA formation (160, 161). A further study using the Ang II-infusion approach in Apoe^−/−^ mice identified a “fibrocyte” cell cluster (Cd34^+^ and Col1a2^+^) which was more prevalent compared to control aorta, and enriched for Mmp2, Mmp3, Mmp14, and Mmp23 (162). Utilising the elastase-induced mouse AAA model and examining the infrarenal abdominal aorta, Mmp9 and Mmp4 were identified as enriched genes across multiple monocyte/macrophage subpopulations, with Mmp9 highly expressed in a specific subpopulation termed “aortic-resident” and characterised through expression of Cx3cr1 and Flt3, which decreased proportionally in elastase-treated mice (163). No differentially expressed MMPs were identified in single-cell RNA sequencing on mouse AAA tissues from the perivascular CaCl_2_ application mouse model (164).

### Role of MMPs and TIMPs in animal models of AAA

Multiple proof-of-principle animal studies have been conducted to determine the contribution of specific MMP and TIMP family members to AAA formation (Table 1). A deleterious role for MMP-2 in AAA formation was proposed based upon reduced aortic diameter in mice with global Mmp2-deficiency using the CaCl_2_-application model (118). The adverse involvement of MMP-2 was attributed to heightened VSMC production as intravenous administration of peritoneal macrophages from wild-type mice failed to reverse the phenotype (118). Assessment of spontaneous AAA formation in long-term high fat-fed Apoe^−/−^ mice with and without Mmp3-deficiency revealed increased presence of aneurysms in Mmp3 wild-type mice, as characterised by elastin fragmentation, aortic wall thinning, and rupture of medial elastin fibres (119). Mice harbouring deletion of Mmp-7 displayed reduced VSMC proliferation and apoptosis numbers within Ang II-induced AAAs compared to wild-type controls, however this did not translate to a difference in AAA expansion or severity (120). Two independent studies demonstrated absence of MMP-9 suppressed aneurysm development in the CaCl_2_-application model (118) and elastase-induced AAAs (121). In addition, both studies utilised a bone marrow transplant technique to suggest that macrophage-derived MMP-9 underlies the induction of AAA in both models (118, 121).

Multiple reports utilising an array of mouse and rabbit models have yielded conflicting findings regarding the role of MMP-12 in AAA development. Mmp12 deficiency attenuated AAA development allied with reduced macrophage recruitment in the peri-aortic CaCl_2_-application model (122), and a mouse model of combined Ang II-infusion and TGF-β neutralising antibody treatment (66). Further supporting the proposition that MMP-12 promotes AAA formation, medial elastin fragmentation and aortic dilation were increased in transgenic rabbits over-expressing MMP-12 in macrophages and subjected to carrageenan-induced AAA, compared to wild-type rabbits (125). However, no effect of Mmp12 knockout on AAA formation was observed in the elastase-perfusion mouse model (121). Conversely, the development of AAAs was reported to have accelerated in two different hypercholesterolaemic mouse models (Apoe-deficient mice or mice over-expressing PCSK9) (124). Additionally, it was proposed that the protective effects were through macrophage expression of MMP-12 suppressing complement activation and subsequent neutrophil infiltration, as macrophage-restricted Mmp12-deficeincy mirrored global Mmp12-deficeincy effects on AAA (124). Aortic dilation was reduced in Mmp13-deficient mice subjected to elastase infusion compared to wild-type animals, suggesting MMP-13 promotes AAA formation (126). An adverse role for MMP-14 in aneurysm progression has been suggested given bone marrow transplantation of Mmp14-deficient macrophages into wild-type mice prevented AAA development in the peri-aortic CaCl_2_-application model (127). Although in opposition, a transgenic mouse harbouring a point mutation in the cytoplasmic domain MMP-14 (Y573D) which abrogates its signalling function without affecting its proteolytic activity, protected from Ang II-induced AAA development (128). Finally, a beneficial effect for MMP-17 was proposed given that lack of Mmp17 resulted in increased susceptibility to ang II-induced AAA expansion, although incidence was unaffected (129).

Focussing on the endogenous MMP inhibitors, TIMPs are largely considered as protective during aneurysm formation, development, and rupture through combatting MMP proteolytic activity. Indeed, deletion of TIMP-1, in both wild type and Apoe^−/−^ mice increased AAA development (130). Moreover, Timp1 knockout, in an elastase-induced model of AAA, resulted in increased aortic diameter and augmented loss of medial elastin (131). Hence, overexpression of TIMP-1 in a rat model of guinea-pig xenograft-induced AAA reduced elastin degradation together with aneurysm formation and rupture (132). On the contrary, the genetic deletion of Timp2 in in the peri-aortic CaCl_2_-application model, suppressed aortic expansion compared to wild type controls with no effect upon medial elastin fragmentation, despite a reduction in MMP-2 activity in Timp2-deficient mice (133). The role of TIMP-3 in the formation of AAA has been explored in the non-atherosclerotic and atherosclerotic Ang II-induced AAA mouse model, with both approaches demonstrating Timp3-deficiency adversely affected vascular remodelling through increased inflammation and proteolytic activity, consequently contributing to reduced collagen and elastin content (32, 134), highlighting the protective role of TIMP-3 on AAAs. No animal study addressing the role of TIMP-4 in AAA development and rupture has been conducted at present.

### MMP pharmacological intervention in animal models of AAA

The accumulating human histological analysis alongside complimentary animal studies provide robust proof-of-principle evidence for dysregulated MMP expression and activity actively contributing to AAA formation and progression. Broad-spectrum targeting of aberrant MMP activity has been explored as a therapeutic strategy to limit the development of experimental AAAs in numerous rodent models (Table 2). Researchers have deployed tetracycline derivatives as non-specific MMP inhibitors alongside other pleiotropic compounds with established MMP-inhibitory capacity. Doxycycline, a broad-spectrum antibiotic, and tetracycline analogue, harbours the ability to non-specifically inhibit MMP activity and expression, as demonstrated in AAA explants and cultured VSMCs (181). In the rat elastase-perfusion AAA model, doxycycline administration was shown to suppress aortic dilation, alongside decreased incidence of AAA formation and medial elastin degradation, and inhibited MMP-9 expression/activity (165, 166, 167). Comparable beneficial effects of doxycycline treatment were detected in mice with established elastase-induced AAAs (168) or during their development (121). Doxycycline treatment induced equivalent effects within a rat aortic ligature model of AAA (176), and in rats where AAAs were stimulated through application of thioglycolate and plasmin (175). Aortic dilation was equally dampened in the mouse peri-aortic CaCl_2_-application AAA model, which was associated with decreased MMP activity (169, 170). Doxycycline was also shown to diminish aortic expansion and inflammation within Ang II-induced AAAs from wild-type mice (171), with reduced AAA development also observed in Apoe^−/−^ mice but with mixed effects upon MMP expression and activity (172, 173). Lastly, AAA incidence and severity were reduced in Ldlr^−/−^ mice infused with Ang II alongside prolonged high fat feeding, although atherosclerotic burden within the aorta was unaffected (174).

**Table 2:** Results of *in vivo* animal studies evaluating the effects of modulating non-specific MMP activity on abdominal aortic aneurysm formation and cellular composition, using broad-spectrum pharmacological inhibitors of MMPs.

Therapeutic	Species	Model	AAA Development; Effects	Ref.
Doxycycline	Rt	Elastase	Reduced; ↓ AD; incidence; elastin deg; MMP-9 activity	(165)
Doxycycline	Rt	Elastase	Reduced; ↓ AD; mac; elastin deg; MMP-9 levels & activity	(166)
Doxycycline	Rt	Elastase	Reduced; ↓ AD; incidence; elastin deg; ↔ inflamm or MMPs	(167)
Doxycycline	Ms	Elastase	Reduced; ↓ AD; incidence	(121)
Doxycycline	Ms	Elastase	Reduced; ↓ AD; incidence; elastin deg	(168)
Doxycycline	Ms	CaCl_2_	Reduced; ↓ AD	(169)
Doxycycline	Ms	CaCl_2_	Reduced; ↓ AD; global MMP activity	(170)
Doxycycline	Ms	Ang II	Reduced; ↓ AD; inflamm	(171)
Doxycycline	Ms	Ang II + Apoe ^−/−^	Reduced; ↓ AD; incidence; MMP-2/-9 activity	(172)
Doxycycline	Ms	Ang II + Apoe ^−/−^	Reduced; ↓ AD; incidence; ↔ MMP expression	(173)
Doxycycline	Ms	Ang II + Ldlr ^−/−^	Reduced; ↓ incidence; severity; ↔ athero burden	(174)
Doxycycline	Rt	Thio + plasmin	Reduced; ↓ AD; elastin deg; MMP-9 activity; ↔ inflamm	(175)
Doxycycline	Rt	Aortic ligature	Reduced; ↓ AD; incidence; MMP-2/9 activity; inflamm	(176)
Hydroxamic acid	Rt	Elastase	Reduced; ↓ AD; elastin deg; inflamm	(177)
Hydroxamic acid	Rt	Elastase	Reduced; ↓ AD; incidence; fibrotic resp; ↔ inflamm	(178)
Hydroxamic acid	Rt	CaCl_2_	Reduced; ↓ AD; incidence; elastin deg; MMP activity	(179)
Hydroxamic acid	Ms	Ldlr ^−/−^	Reduced; ↓ elastin deg; ectasia; ↔ MMPs or athero	(180)

↓, decreased; ↑, increased; ↔, no change.

Ms, mouse; Rt, rat; BMT, bone marrow transplantation; Apoe, apolipoprotein E; Ldlr, low-density lipoprotein receptor; Ang II, angiotensin II; CaCl2, calcium chloride; thio, thioglycolate; AD, aortic dilation; deg, degradation.

Batimastat, a synthetic peptide also known as BB-94, is a hydroxamic acid-based broad-spectrum inhibitor of MMPs, and it was demonstrated that systemic administration of BB-94 reduced aortic dilatation and medial elastin fragmentation in elastase-induced AAAs in rats, and also suppressed inflammatory cell infiltration (177). Moreover, targeted delivery and inhibition achieved through delivery of elastin antibody-conjugated BB-94-loaded nanoparticles retarded AAA MMP activity, elastin degradation, calcification, and aneurysmal development in a peri-aortic CaCl_2_-application rat model (179). An alternative hydroxamate-based MMP antagonist, RS 132908, also suppressed aortic dilatation in a rat intraluminal elastase-perfusion AAA model, preserving medial elastin integrity and exerting a medial pro-fibrotic response, although no effect on inflammation was observed (178). Furthermore, CGS 27023A, a similar hydroxamic acid MMP inhibitor, reduced elastin degradation and medial degeneration within the atherosclerotic abdominal aortae of hypercholesterolaemic Ldlr^−/−^ mice (180).

### MMP inhibitors in clinical trials and limitations

The positive response to doxycycline treatment in animal models of AAA resulted in the undertaking of several clinical trials being carried out globally. Doxycycline treatment for 3-months in patients with small AAAs (diameter <5.5 cm) significantly reduced AAA expansion at 6- to -12 and 12- to 18-month follow-up periods, in a double-blind, placebo-controlled, randomised clinical trial (182). Moreover, longer-term (6-month) doxycycline treatment reduced MMP-9 plasma levels as well as maximum aortic diameter in EVAR patients within a randomised, placebo-controlled study (183). Short-term doxycycline treatment (2-weeks) increased TIMP-1 and reduced MMP-8, MMP-9, MMP-3, and MMP-25 expression, resulting in a decrease of inflammatory cell accumulation within diseased aorta of AAA patients (184, 185). Nevertheless, conflicting results in other studies have raised some concerns. A phase II study indicated no changes in AAA diameter after 6-months of doxycycline treatment, despite an observed reduction in MMP-9 plasma levels (186). Additionally, in a randomised, placebo-controlled, and double-blind study, doxycycline treatment did not affect expression or activity of any of the MMPs and TIMPs assessed (187). A further randomised, placebo-controlled, double-blind study utilising a larger patient cohort, demonstrated that long-term doxycycline treatment (18-months) associated with increased AAA growth, and did not impact the requirement for AAA repair or time to surgical repair (188). Lastly, a parallel, two-group randomised clinical trial across 22 US clinical centres demonstrated doxycycline had no effect upon the growth of small infrarenal AAAs at two-years (189). Accordingly, the ambivalent outcomes have not fostered the therapeutic adoption of doxycycline as a treatment for reducing the expansion of AAAs in humans, with ambiguous outcomes with broad-spectrum MMP inhibitors suggesting the selective targeting of individual MMPs is desirable.

Although an important role for MMPs in AAAs is evident from the published literature, a major limitation of targeting these proteases clinically rests in the difficulty in designing highly selective MMP inhibitors that remain effective *in vivo*. MMPs govern a variety of biological processes that can exert beneficial and detrimental effects upon the pathophysiology of AAAs alongside physiological processes throughout the body, hence the ongoing attempts to pharmacologically target select MMPs using small organic inhibitors. However, due to the high structural homology of MMP family members, early-stage MMP inhibition has commonly failed as a therapeutic strategy in clinical trials. Such failures have been attributed to the poor specificity of the compounds, in part due and their broad inhibitory profile, resulting in effects on multiple MMPs which exert divergent properties on differing matrix proteins and disparate cell types. Additionally, shared substrates and cross-reactivity between MMP family members (alongside ADAMs and ADAMTSs) remains a continual obstacle, that requires novel strategies which can distinguish between the activities of separate MMPs. Next generation MMP inhibitors must be highly selective, specific, and preferably inhibit a single MMP function, and would benefit from cell-type directed delivery advances to negate off-target effects.

## Conclusions and future prospective

In summary, there is considerable evidence supporting a direct role for dysregulated MMP expression and subsequent activity to the formation and progression of AAAs. Evidence demonstrates that destruction of elastin and collagen can be limited or reversed with MMP inhibition, while animal studies have confirmed the anomalous role and function of individual MMPs during AAA development. Accordingly, modifications to restore or over-express TIMP family members have proven to effectively suppress MMP activity and prevent the development and progression of AAAs in animal models. However, the inability of broad-spectrum MMP inhibition to successfully provide clinical translation has led to renewed interest in the elaboration of MMP inhibitors that exhibit narrow specificity, as has been successfully demonstrated in atherosclerosis studies utilising inhibitors restricted to the targeting of MMP-12 (101) or MMP-13 (190). Indeed, the direct association of heightened macrophage infiltration and elastin degradation to the pathogenesis of AAAs, in marriage with the central role of MMP-12 to both processes suggests that perturbing the resulting medial wall inflammation and destruction of elastin could be achieved through inhibition of MMP-12 activity. Therefore, future studies focussing on the development of MMP-12 specific inhibitors as a potential therapeutic for AAA would appear desirable. However, evaluating the efficacy of MMP inhibition while also identifying patients who will benefit most from associated therapeutics would also be advantageous. Accordingly, there is a keen interest to develop imaging approaches to identify MMP activity within AAAs. Two recent studies demonstrating how MMP inhibitors or substrates can be engineered to permit visualisation of MMP activity within AAAs (191, 192) may therefore facilitate prognosis and potential treatment stratification. So, notwithstanding the limitations associated with translating findings from animal models to human AAA, the reviewed studies should encourage renewed motivation for future clinical trials of selective MMP inhibitors for the treatment of existing AAAs. Furthermore, new insight into the spatial and temporal expression and activity of MMPs and TIMPs, alongside cell-type expression patterns, may aid the design of future therapeutics.
